# Molecular magneto-ionic proton sensor in solid-state proton battery

**DOI:** 10.1038/s41467-022-34874-6

**Published:** 2022-11-17

**Authors:** Yong Hu, Zipeng Guo, Yingjie Chen, Chi Zhou, Yuguang C. Li, Shenqiang Ren

**Affiliations:** 1 Department of Mechanical and Aerospace Engineering, University at Buffalo, The State University of New York, Buffalo, NY 14260 USA; 2 Department of Industrial and Systems Engineering, University at Buffalo, The State University of New York, Buffalo, NY 14260 USA; 3 Department of Chemistry, University at Buffalo, The State University of New York, Buffalo, NY 14260 USA; 4 Research and Education in Energy Environment & Water Institute, University at Buffalo, The State University of New York, Buffalo, NY 14260 USA

**Keywords:** Magnetic materials, Ferroelectrics and multiferroics, Sensors and biosensors

## Abstract

High proton conductivity originated from its small size and the diffusion-free Grotthuss mechanism offers immense promise for proton-based magneto-ionic control of magnetic materials. Despite such promise, the realization of proton magneto-ionics is hampered by the lack of proton-responsive magnets as well as the solid-state sensing method. Here, we report the proton-based magneto-ionics in molecule-based magnet which serves as both solid-state proton battery electrode and radiofrequency sensing medium. The three-dimensional hydrogen-bonding network in such a molecule-based magnet yields a high proton conductivity of 1.6 × 10^−3^ S cm^−1^. The three-dimensional printed vascular hydrogel provides the on-demand proton stimulus to enable magneto-ionics, where the Raman spectroscopy shows the redox behavior responsible for the magnetism control. The radiofrequency proton sensor shows high sensitivity in a wide proton concentration range from 10^−6^ to 1 molar under a low working radiofrequency and magnetic field of 1 GHz and 405 Oe, respectively. The findings shown here demonstrate the promising sensing application of proton-based magneto-ionics.

## Abstract (web summary)

Ionic control of magnetism promises ultralow-field sensor, but current physical realizations of proton-based magneto-ionic sensor are limited due to the lack of effective solid-state sensing methods. Here, authors report magneto-ionics-based proton sensing under low working radiofrequency and magnetic fields.

## Introduction

**Fig. 1:**
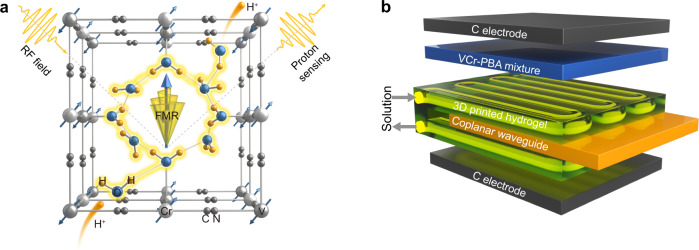
Molecular magnet-based ionics for proton sensing. **a** Schematic figure for magneto-ionic-based proton sensing. The VCr-PBA has vacancy and hydrogen-bonding networks (highlighted in yellow), allowing for rapid reversible proton transfer through the diffusion-free Grotthuss mechanism. The incident RF field could couple with the molecular magnetization through ferromagnetic resonance (FMR). The RF power is absorbed by the precessing magnetization (Larmor precession) of the molecular based-magnet, resulting in the loss of power of the outgoing RF field. The proton sensing is realized by monitor the outgoing RF field power. **b** Structure of the magneto-ionic based proton sensing devices, including carbon paper coated with VCr-PBA mixture [70 wt.% compounds 1 + 10 wt.% Polyvinylidene fluoride (PVDF) + 20 wt.% carbon black] (working electrode), 3D printed hydrogel with channels for the solution to flow in/out, coplanar waveguide, and carbon paper (counter electrode).

Magneto-ionic control of magnetic materials is based on the ionic motion and electrochemical reactions activated by external voltage^1,2^. In recent years, we have witnessed the successful ion control of magnetic properties for inorganic and molecule-based magnets from the strong coupling between ion and spin polarization, offering immense promise for developing magneto-ionic-based sensors^1–7^. In this context, molecular materials with porous and vacancy networks particularly attract significant interests due to its stimuli responsive magnetism. For example, lithium control of magnetism in molecule-based magnet has been integrated with rechargeable lithium-ion batteries for real-time state-of-charge estimation^7^. However, alkali metal ions typically show a limited diffusion rate in molecular materials due to its relatively large ionic radii. Compared with lithium ions, proton could become one of the effective ions to control magnetism, due to its small size and high ionic conductivity originating from the diffusion-free Grotthuss mechanism^8,9^. Despite such promise, proton-based magneto-ionic sensors have been limited for the selection of molecular magnet candidates which can be proton-responsive and proton-solution (i.e. hydrogel) compatible. In addition, a solid-state radiofrequency (RF) FMR based sensing method is necessary for the incorporation of a magneto-ionic-based sensor within a proton-based functional device, such as a proton rechargeable battery. The magnetic characterization methods, like vibrating-sample magnetometer, superconducting quantum interference device magnetometer, and magneto-optic Kerr effect, could provide in-situ magnetic measurements. However, the required vibration or optical setup makes them challenging for the integration with the proton battery. To overcome these challenges, we select molecule-based magnets, magnetic Prussian blue analogues A_*x*_M[M’(CN)_6_]_*y*_·*z*H_2_O (A, alkali metal; M, M’, transition metal; hereafter denoted as MM’-PBA), due to their structural vacancy and hydrogen bonding networks to induce fast proton transfer through the diffusion-free Grotthuss mechanism (Fig. 1a)^8–13^. Such a high proton conductivity in molecule-based magnets also makes them an excellent electrode for building proton batteries with high-rate capability and long cycle life^4^. The hydrogel, high water content of more than 80%, promises the solid-state reservoir of proton source with the capability of rapidly printing complex geometries for three-dimensional (3D) functional vascular structures compatible with solid-state RF FMR-based detection (Fig. 1b)^14,15^.

Here, we report a proton-responsive magneto-ionic material, room-temperature ferrimagnetic Prussian blue analogues (PBA) of (V[Cr(CN)_6_]_0.85_·4.2H_2_O, VCr-PBA) (Supplementary Figs. 1 and 2), for the protonation-induced magnetism control (Fig. 1a). VCr-PBA consists of the vacancy networks, [Cr(CN)_6_] and the V ions coordinated to ligand water molecules, and zeolitic water molecules in the vacancy sites^16,17^. The 3D hydrogen-bonding network between the ligand and zeolitic water molecules serves as the ion migration channels, enabling a high proton conductivity of 3.65 × 10^−4^ S cm^−1^. Raman spectroscopy shows the redox behavior of transition metal ions in VCr-PBA responsible for the magnetism control, where the printed solid-state hydrogel vascular network serves as the proton reservoir. The RF-based FMR sensing enables the real-time detection of a wide proton concentration range from 10^−6^ to 1 molar (M) with a low working RF and magnetic field of 1 GHz and 405 Oe, respectively. The magneto-ionic-based proton concentration measurement allows a solid-state hydrogel-based proton sensing which is complementary to conventional electronic-based approaches, such as conductivity measurement.

## Results and discussion

### Electrochemical analysis

**Fig. 2:**
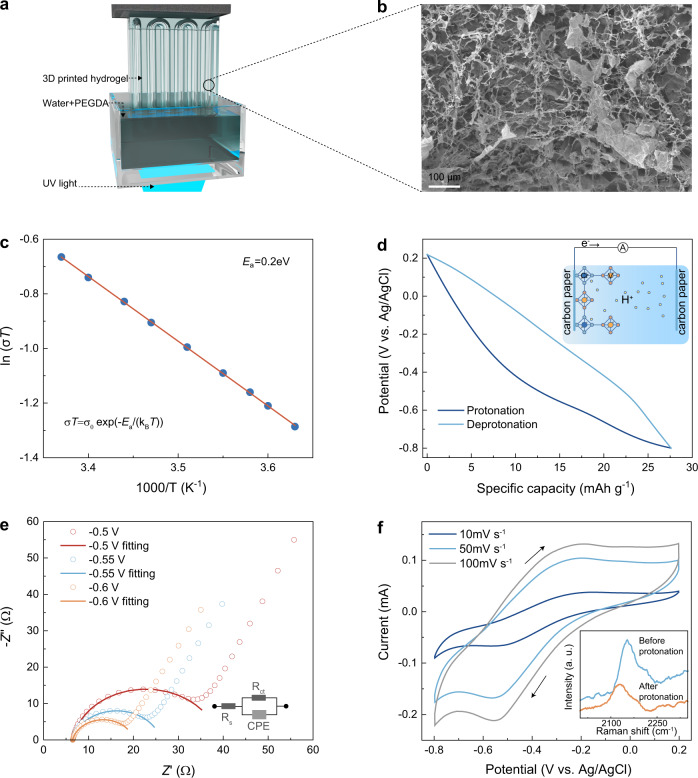
Electrochemical analysis for 3D printed hydrogel and VCr-PBA. **a** Schematic diagram of the 3D printed hydrogel for the magneto-ionic proton sensor. The UV light used has a wavelength of 385 nm and the control of image projection was achieved through a dynamic micromirror device. **b** SEM image for the PEGDA scaffold in the 3D printed hydrogel. **c** The plot of ln(σT) vs. 1000/T for VCr-PBA. *σT* = *σ*_0_ exp(-*E*_a_/(*k*_B_*T*)). σ refers to proton conductivity. E_a_ is the activation energy. σ_0_ represents the pre-exponential factor, and k_B_ represents the Boltzmann constant. **d** The specific capacity-dependent potential during protonation and deprotonation process for VCr-PBA under 0.36 A g^−1^ rate. Specific capacity=current × time/mass. **e** Nyquist plots for VCr-PBA at various protonation levels. Z’ is the real part and Z” is the imaginary part. R_s_ represents the high-frequency series resistor, R_ct_ represents the charge-transfer resistance. CPE is the constant phase element that represents surface capacitance. **f** Cyclic voltammetry curve for VCr-PBA at 10, 50, and 100 mV s^−1^. Inset shows the protonation-induced change in the Raman spectra of VCr-PBA. Source data are provided as a Source Data file.

Figure 1b shows the scheme for VCr-PBA-based proton sensing. In a proton-based magneto-ionic device as compared with the proton-based battery, the H_2_SO_4_ solution (electrolyte) is stored in the hydrogel which also acts as a separator^18,19^. Fig. 2a and Supplementary Movie 1 show the printed vascular hydrogel network obtained by continuous stereolithography (SLA)^15^. The poly(ethylene glycol) diacrylate (PEGDA)-cross-linked hydrogels (Fig. 2b) show a porous cellular structure with macropores of several micrometers in diameter^20^. These connected pores in hydrogel store the injected proton medium and provide the pathways for transporting protons to electrodes^14^. We investigate the proton conductivity of VCr-PBA at different temperatures. As shown in Fig. 2c, a high proton conductivity of 1.6 × 10^−3^ S cm^−1^ is achieved at 293 K, suggesting VCr-PBA as a superionic conductor (σ > 10^−4^ S cm^−1^)^4^. The low activation energy of 0.2 eV suggests the proton transfer through the diffusion-free Grotthuss mechanism in the hydrogen-bonding networks connected by zeolitic and ligand water molecules^8^. The electrochemical properties of VCr-PBA during protonation/deprotonation are investigated by the galvanostatic discharge/charge measurements. Protons can diffuse through the hydrogel pores and intercalate into VCr-PBA during the discharging process. The charging state reverses the protonation process. As shown in Fig. 2d, the discharging capacity reaches 27.5 mAh g^−1^ under 0.36 A g^−1^ in the ferrimagnetic VCr-PBA. Cycle number-dependent specific capacity (Supplementary Fig. 3) shows that the protonation/deprotonation process is reversible.

To provide an understanding of the protonation kinetics of VCr-PBA, electrochemical impedance spectroscopy (EIS) measurements are performed at various discharging levels. As shown in the inset of Fig. 2e, an equivalent electrical circuit model is adopted to fit the EIS data^21^. The R_s_, representing ohmic impedance comprised of ion exchange membrane resistance, contact resistance, solution resistance, and electrode resistance, maintains constant at different protonation levels. The charge transfer resistance (R_ct_) across the electrode-electrolyte interface shows a decreasing tendency as the protonation level increases. Upon protonation to −0.5, −0.55, and −0.6 V, the R_ct_ decreases to 32.9, 19.6, and 13.6 Ω, respectively, suggesting that protonation makes proton readily intercalate into the lattice. Cyclic voltammetry (CV) measurements for VCr-PBA are also performed at various rate to reveal the protonation and deprotonation process. As shown in Fig. 2f, two current peaks are observed at around −0.55 V and −0.2 V vs. Ag/AgCl, which can be ascribed to the reduction and oxidation of [Cr^III^(CN)_6_]^3−^ to [Cr^II^(CN)_6_]^4−^ and [Cr^II^(CN)_6_]^4−^ to [Cr^III^ (CN)_6_]^3−^, respectively^22^. The redox process for [Cr^III^(CN)_6_]^3−^ could be formulated as: [Cr^III^(CN)_6_]^3−^ + xe^−^ + xH^+^ ⇌ (1-x)[Cr^III^(CN)_6_]^3−^ + x{H[Cr^II^(CN)_6_]}^3−^. (x = 0.3 for the protonation of 27.5 mAh g^−1^). The reduction of [Cr^III^(CN)_6_]^3−^ can also be supported by the change in the C≡N bonds which are connected to Cr atoms^4^. As shown in the inset of Fig. 2f, the ex-situ Raman spectroscopy shows that the C≡N bond at 2108 cm^−1^ shifts to a lower wavenumber of 2075 cm^−1^ after protonation as a result of the reduction-induced C≡N bonds deformation.

### Magneto-ionic control of magnetism

**Fig. 3:**
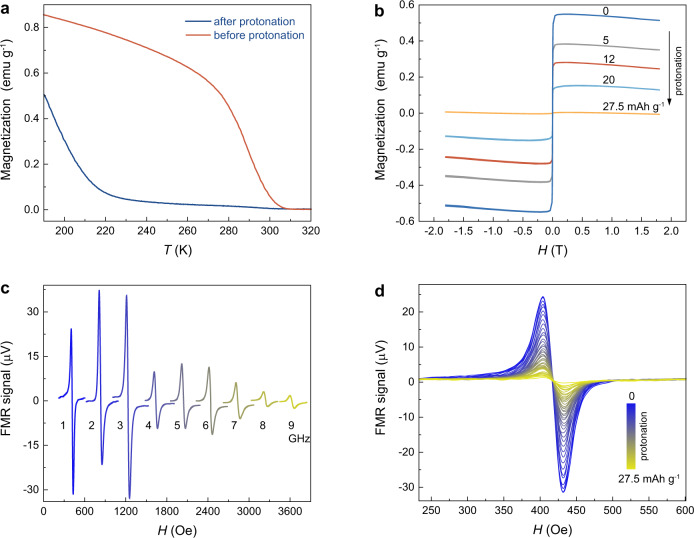
Proton control of magnetism for VCr-PBA. **a** Temperature dependence of magnetization of VCr-PBA before and after protonation. **b** Magnetic hysteresis loops measured at room temperature for VCr-PBA at various protonation levels. **c** The FMR spectra measured at room temperature and RF conditions for VCr-PBA. **d** Room-temperature FMR spectra under 1 GHz for VCr-PBA at different protonation levels. Source data are provided as a Source Data file.

As shown in the electrochemical analysis, protonation and deprotonation processes, enabled by the hydrogen bonding networks, induce the electronic structure change in VCr-PBA. Therefore, we further study its magnetic response during the protonation. Temperature dependence of magnetization measurements are examined in Fig. 3a. Before protonation, VCr-PBA shows a ferrimagnetic to paramagnetic transition with a critical temperature (*T*_c_) of 305 K^12,23,24^. After the proton is inserted into the lattice at a level of 27.5 mAh g^−1^, the *T*_c_ of VCr-PBA shifts to a lower temperature point of 220 K, suggesting the protonation control of magnetism. The magnetic properties for VCr-PBA at different protonation levels are further studied using room-temperature magnetic hysteresis measurements. As shown in Fig. 3b, VCr-PBA shows a saturated magnetization of 0.55 emu g^−1^ before protonation. As the protonation level goes up to 27.5 mAh g^−1^, the saturated magnetization shows a decreasing tendency to 0.01 emu g^−1^. The decrease in magnetization after protonation could be resulted from the transition from high-spin [Cr^III^(CN)_6_]^3−^ to low-spin [Cr^II^(CN)_6_]^4−^ states^4^. After demonstrating the proton control of magnetism, we further study the FMR-based measurement for revealing the proton-dependent magneto-ionics in VCr-PBA, which is essential for the development of a molecule-based magneto-ionic proton sensing device. The FMR derivative spectra for VCr-PBA in the sensing device (Fig. 1b) are measured with the dependence of RF frequencies and magnetic fields (Fig. 3c). It should be noted that the FMR signal is large at low RF frequency, which is mainly resulted from high S_21_ parameter in the device. A higher S_21_ parameter would generate a large RF field around VCr-PBA, which would bring a stronger FMR signal compared with the lower S_21_ parameter. The S_21_ parameter is about −6 dB at 1 GHz and reduces to −55 to −60 dB for the frequency from 5 to 9 GHz (Supplementary Fig. 4). As shown in Supplementary Fig. 5a, the resonance magnetic field increase linearly with the resonant RF frequency, suggesting its ferrimagnetic behavior. It is noted that the VCr-PBA exhibits homogeneous magnetic properties, evidenced by its narrow peak-to-peak width of ~30 Oe at 1 GHz. For the RF-based magneto-ionic proton sensing, the FMR signal and protonation level should be bijective. Thus, we further perform in-situ FMR measurements at constant magnetic and RF fields during the protonation process to reveal the relationship between protonation and magnetism. At a 1 GHz microwave source, a low resonant magnetic field of 420 Oe is obtained, which is also an advantage for a low-field operation environment. Thus, the in-situ FMR measurement during protonation is performed at a 1 GHz RF field. As shown in Fig. 3d, the FMR signal is pronounced before protonation. The FMR signal shows a decreasing tendency as the protonation level is increased (a diminished intensity obtained at a protonation level of 27.5 mAh g^−1^), promising for the applications as proton sensor.

### Magneto-ionic based proton sensing

**Fig. 4:**
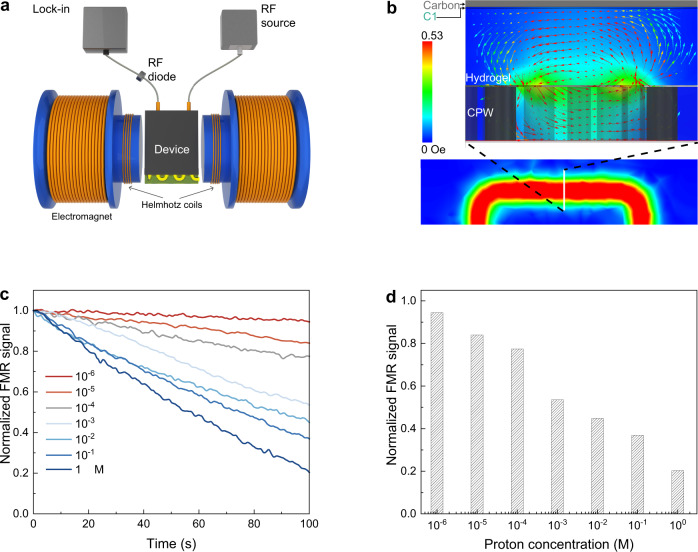
Magneto-ionic based proton sensing. **a** Proton sensing setup. **b** Simulation results for the magnetic field distribution from the RF field in the proton sensing device (Supplementary Movie 2). **c** Time-dependent normalized FMR signal under different proton concentrations. A constant bias voltage of −0.7 V is applied to the device during the measurement. **d** Proton concentration-dependent normalized FMR signal at 100 s. Source data are provided as a Source Data file.

The proton control of magnetism in VCr-PBA enables the development of the RF-based proton sensor (Fig. 4a). Microwave is generated from the RF source connected to the coplanar waveguide (CPW) (Supplementary Fig. 6), where the RF field and a static magnetic field are applied as the resonance field source based on the FMR resonance condition shown in Fig. 3c. To increase the signal-to-noise ratio, an alternating current (AC) magnetic field is also applied for the lock-in based detection. It is noted that RF-based magnetic resonance sensing is scalable based on the semiconductor chips^25^. The microwave absorption in VCr-PBA is measured via a phase-sensitive detection using an RF diode (Schottky detector) connected to the CPW. The detected FMR signal is the derivative of microwave power absorption with respect to external magnetic field. The Ansys HFSS (high-frequency structure simulator) simulation shows that the CPW can deliver a strong electromagnetic field around the sensing region (between the hydrogel and top carbon paper electrode, shown in Fig. 4b and Supplementary Movie 2). A constant voltage of −0.7 V is applied to the working electrode. The concentration-dependent deprotonation study (Supplementary Fig. 7) shows that decreasing the proton concentration would reduce the discharging current which might come from the higher electrolyte resistance at lower proton concentration (Supplementary Fig. 8). It is noted that the magnetism of VCr-PBA is highly related to the protonation level, suggesting the protonation concentration could also be detected from the FMR. As shown in Fig. 4c, the FMR signal decreases as the bias voltage is applied to the working electrode. A higher proton concentration shows a fast-decreasing rate of FMR signal, suggesting the detection of proton concentration by reading the FMR signal at a time. As shown in Fig. 4d, the FMR signal at 100 s is plotted against the proton concentration. The normalized FMR signal are 0.94, 0.84, 0.77, 0.54, 0.45, 0.37, 0.20 for 10^−6^, 10^−5^, 10^−4^, 10^−3^, 10^−2^, 10^−1^, 1 M proton, respectively. It is noted that the sensing time could be future optimized by reducing the thickness of the studied electrode layer (38 μm, Supplementary Fig. 9) to a few hundred nanometers^22^.

We demonstrate an all-solid-state magneto-ionic proton sensor based on a molecule-based magnet serving as both solid-state proton battery electrode and radiofrequency (RF) sensing medium. The 3D hydrogen-bonding network between the ligand and zeolitic water molecules provides the ion migration channels, enabling a high proton conductivity of 1.6 × 10^−3^ S cm^−1^. Raman spectroscopy shows the redox behavior of transition metal ions in VCr-PBA during the magnetism control. The RF-based proton sensing device is shown in a wide proton concentration range from 10^−6^ to 1 M with a low working RF and magnetic field of 1 GHz and 405 Oe, respectively. Our study shows that a solution-processable molecule-based magnet is of particular interest for magneto-ionic-based ion sensors.

## Methods

### VCr-PBA and working electrodes preparation

VCr-PBA is obtained by mixing of 360 mg Potassium hexacyanochromate(III) and 240 mg Vanadium(II) chloride in 50 mL deoxygenated distilled water. The reacted suspension is washed three times with deoxygenated water by centrifugation (3340 × g, 10 min) to remove the unreacted ions. The obtained compounds were dried under vacuum for one day to get the dark blue powder sample. The dried sample is put in the nitrogen environment chamber with 100% humidity for post-treatment. Collected: 124 mg of solid. VCr-PBA (70 wt%), graphene (15 wt%), and polyvinylidene fluoride (15 wt%) were first mixed into a slurry and them cast onto carbon fiber paper, and finally dried on the hotplate (333 K) for preparing the working electrode. The thickness, mass loading for working electrode is 38 μm, 2 mg cm^−2^.

### Hydrogel precursor preparation

15 vol% PEGDA MW 700 (Sigma Aldrich) is added to the water. 0.2 wt% lithium phenyl-2,4,6-trimethylbenzoylphosphinate photo-initiator and 0.002 wt.% yellow-colored dye are added to the precursor.

### Lyophilization

The printed hydrogel is transferred to a freezer (253 K) and placed on a cold aluminum plate for 2 h. The frozen samples are freeze-dried using a freeze-drying system (LABCONCO, USA) operating under a 0.015 mBar vacuum. The primary drying process starts from 233 K then ramped to 263 K at a rate of 0.2 K min^−1^ and holds at −10 °C for 10 h. Next, the secondary drying process ramps to 278 K at a rate of 0.5 K min^−1^ and holds for 6 h. Finally, the temperature is elevated to 293 K and held for 2 h to allow completely solvent sublimation.

### Magnetic properties, morphology, optical and thermal characterization

Temperature-dependent magnetization and magnetic hysteresis loops of molecular magnetic materials are measured using a MicroSense EZ7 vibrating sample magnetometer (VSM). The SEM and energy-dispersive X-ray spectroscopy images are obtained using a Hitachi S4000 SEM microscope. Raman spectroscopy was carried out by using a Renishaw inVia Raman microscope (Renishaw, Inc. Hoffman Estates, IL) with a Leica DMLM microscope. Thickness of electrode layer was measured using Tencor Alpha Step 200 stylus profilometer. The thermal analyses are performed in a TA Instruments SDT Q600 thermobalance using nitrogen flow (200 mL min^−1^).

### Ferromagnetic resonance and coplanar waveguide simulation

FMR spectroscopy measurements were performed using a home-built coplanar waveguide setup. A vector network analyzer (Agilent/HP 8719D) is used for providing and detecting RF signal to a coplanar waveguide. A Schottky detector is applied to convert the RF signal into electrical signal. The microwave absorption (S21 parameter) by the molecule-based magnet is measured via a lock-in detection. AC modulation magnetic field is generated by a Helmholtz coil for locking detection. The coplanar waveguide is designed and simulated by using ANSYS high-frequency structural simulator (HFSS).

### Electrochemical and proton conductivity measurements

Protonation and deprotonation for VCr-PBA is performed in a half cell which includes 1 M H_2_SO_4_ electrolyte and as a carbon paper counter electrode. A Squidstat Plus potentiostat (Admiral Instrument, USA) is used for all electrochemical measurements. The device is kept in a chamber filled with nitrogen at room temperature. Cyclic voltammetry measurements are performed at room temperature from 0.2 to −0.8 V (vs. Ag/AgCl). The AC impedance spectra for each electrode are recorded in a frequency range of 0.1 Hz to 100 kHz and AC amplitude of 0.01 V. The VCr-PBA powder was compressed into a pellet with a diameter of 6 mm and a thickness of 1 mm. The measurements are performed at 100% relative humidity as a function of temperature.

### Stereolithography (SLA) printing

The SLA printing process was performed with a custom-built SLA printer, utilizing a bottom-up configuration, as shown in Supplementary Fig. 10.

## Supplementary information

Supplementary Information

Description of Additional Supplementary Files

Supplementary Movie 1

Supplementary Movie 2

## Data Availability

The data generated in this study are provided within the manuscript and Supplementary Information file. Any additional information needed is available from the corresponding author upon request. Source data are provided with this paper.

## Source data

Source Data
