# Supplementary material for: Molecular magneto-ionic proton sensor in solid-state proton battery
Source: Nat Commun. 2022 Nov 17;13:7056. doi: 10.1038/s41467-022-34874-6 (PMC9672057; doi:10.1038/s41467-022-34874-6)
Supplement: Supplementary file 1 — Supplementary Information [file 41467_2022_34874_MOESM1_ESM.pdf]

Supplementary Information for

Molecular magneto-ionic proton sensor in solid-state proton battery

Yong Hu[1], Zipeng Guo[2], Yingjie Chen[3], Chi Zhou[2], Yuguang C. Li[3*], and Shenqiang Ren[1,3,4*]

Affiliations:

[1]Department of Mechanical and Aerospace Engineering, University at Buffalo, The State University of New York, Buffalo, NY 14260, USA

[2]Department of Industrial and Systems Engineering, University at Buffalo, The State University of New York, Buffalo, NY 14260, USA

[3]Department of Chemistry, University at Buffalo, The State University of New York, Buffalo, NY 14260, USA

[4] Research and Education in Energy Environment & Water Institute, University at Buffalo, The State University of New York, Buffalo, NY 14260, USA

*Correspondence to: yuguangl@buffalo.edu; shenren@buffalo.edu

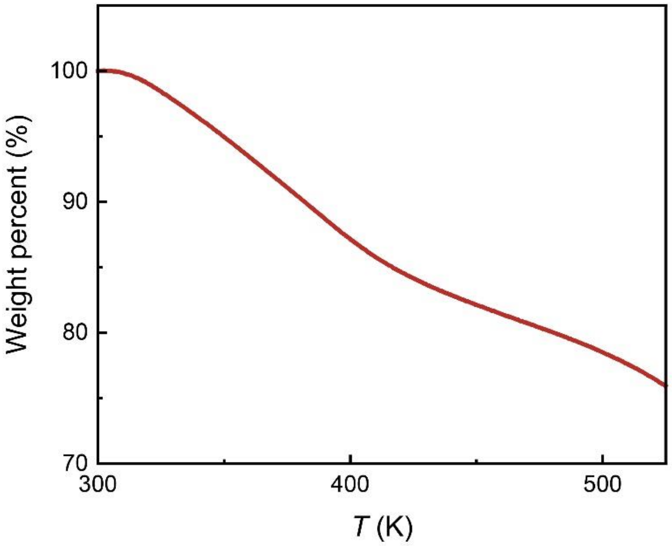

Supplementary Fig. 1. Thermogravimetric analysis measurement for VCr-PBA.

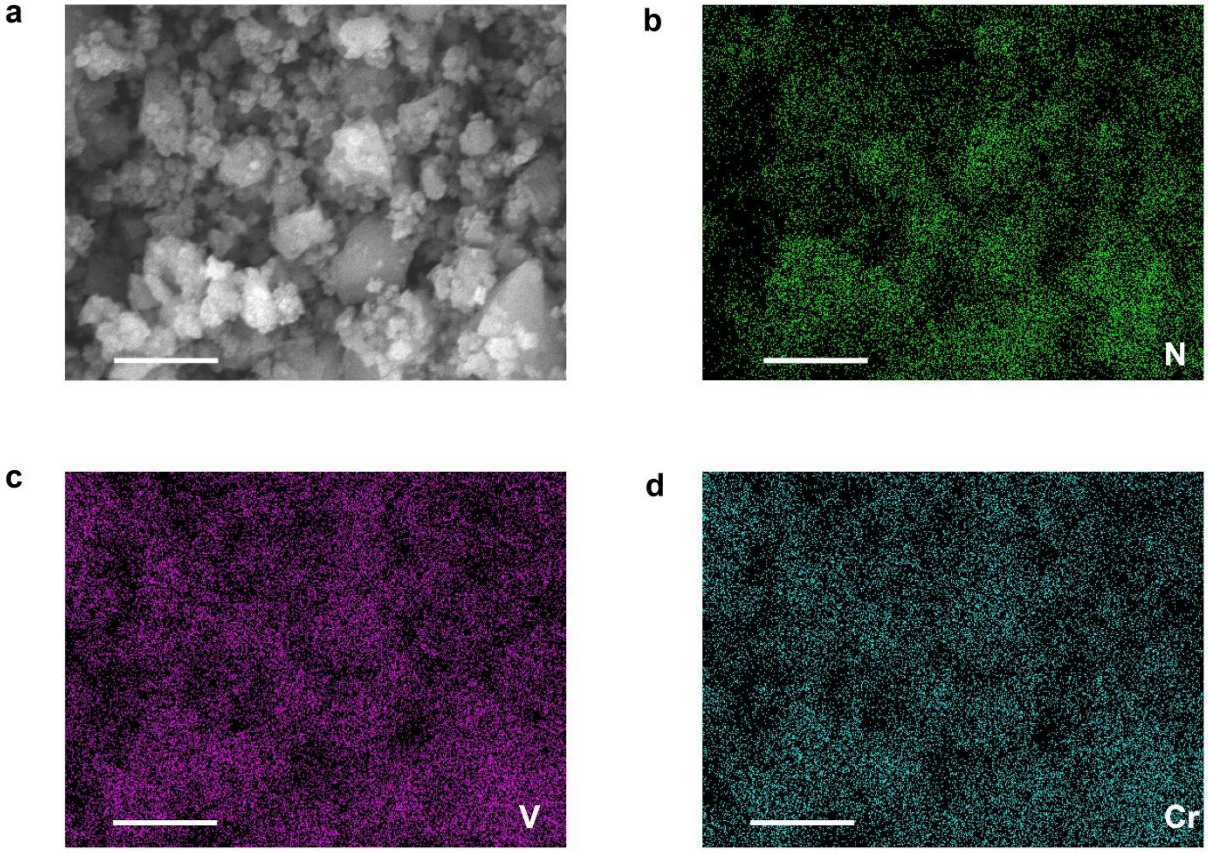

Supplementary Fig. 2. **a**, SEM image for VCr-PBA nanoparticles. EDS mapping for **b**, N, **c**, V, and **d**,Cr elements. The scale bar is 1μm.

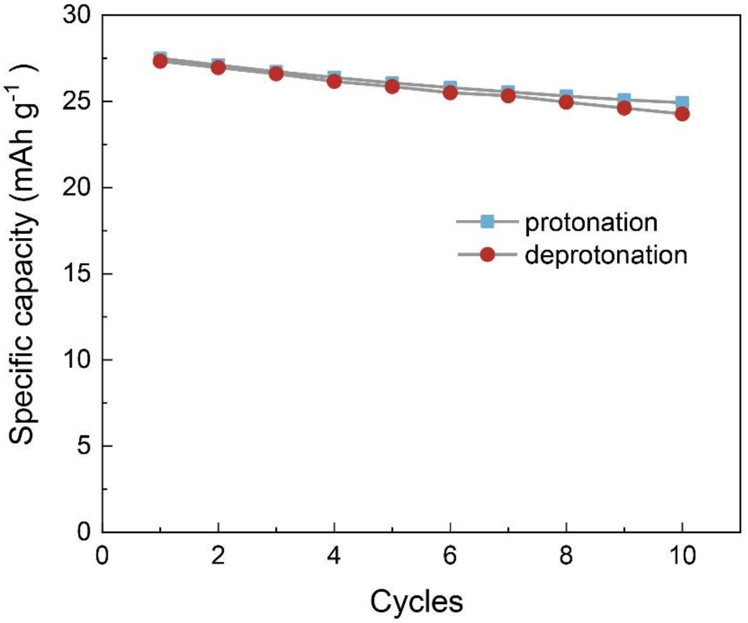

Supplementary Fig. 3. Specific capacity for protonation/deprotonation process under different cycles.

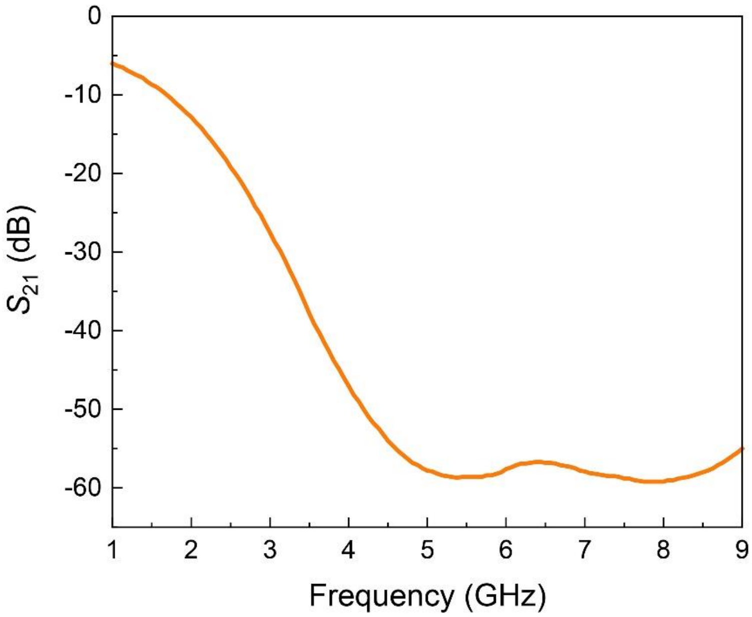

Supplementary Fig. 4. S_{21} parameter of coplanar waveguide (CPW) in the device.

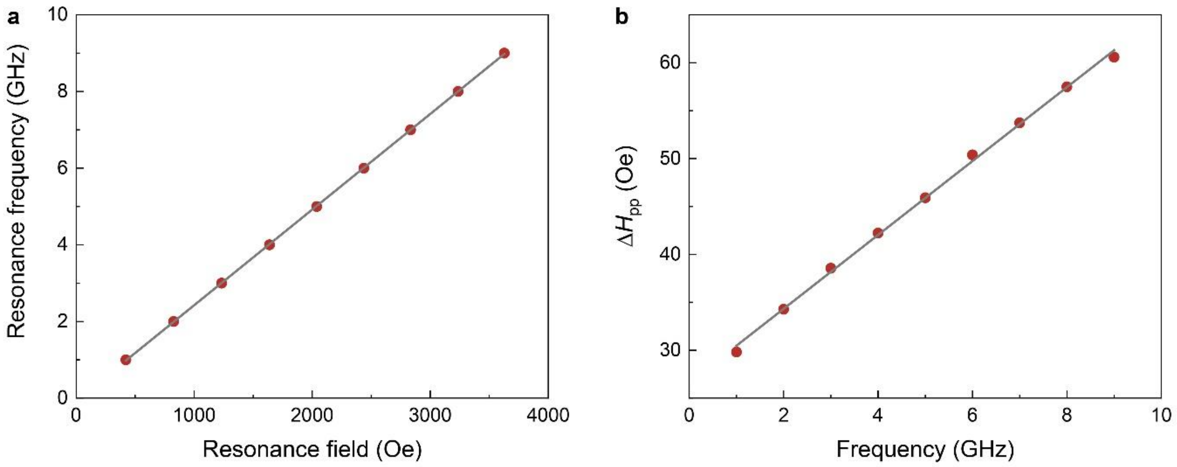

Supplementary Fig. 5. a, Resonance frequency as a function of resonance field for VCr-PBA. **b**, Resonance linewidth (peak to peak, $\triangle H_{pp}$) as a function of frequency for VCr-PBA. Solid lines are the fitting results using $\Delta H(\omega)=\Delta H(0)+\frac{2\alpha\omega}{\sqrt{3}\gamma}$ [1]. ΔH(ω) and ΔH(0) is the frequency dependent and independent peak-to-peak linewidth. α is Gilbert damping constant. γ is the gyromagnetic ratio. ω is the resonance frequency.[1]

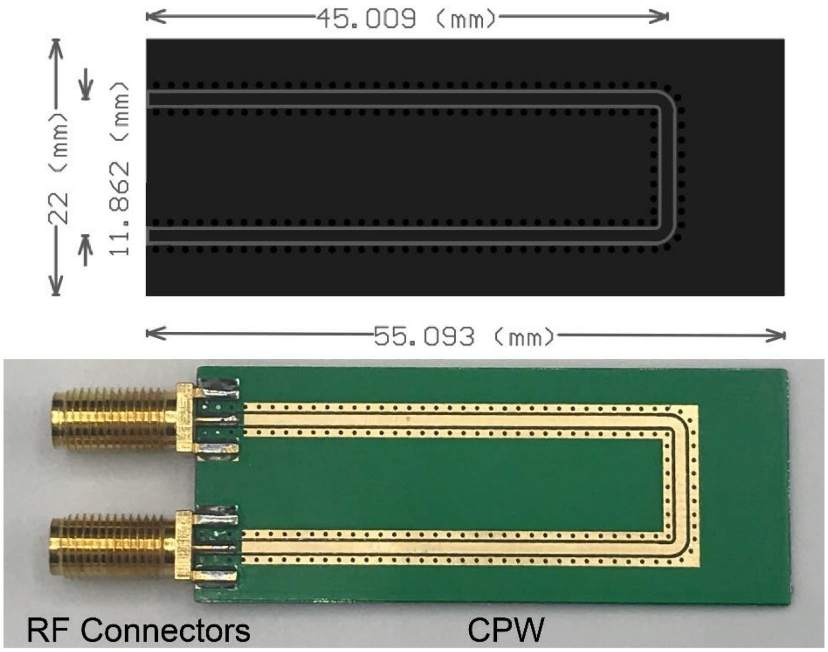

Supplementary Fig. 6. Optical image and layout for designed CPW.

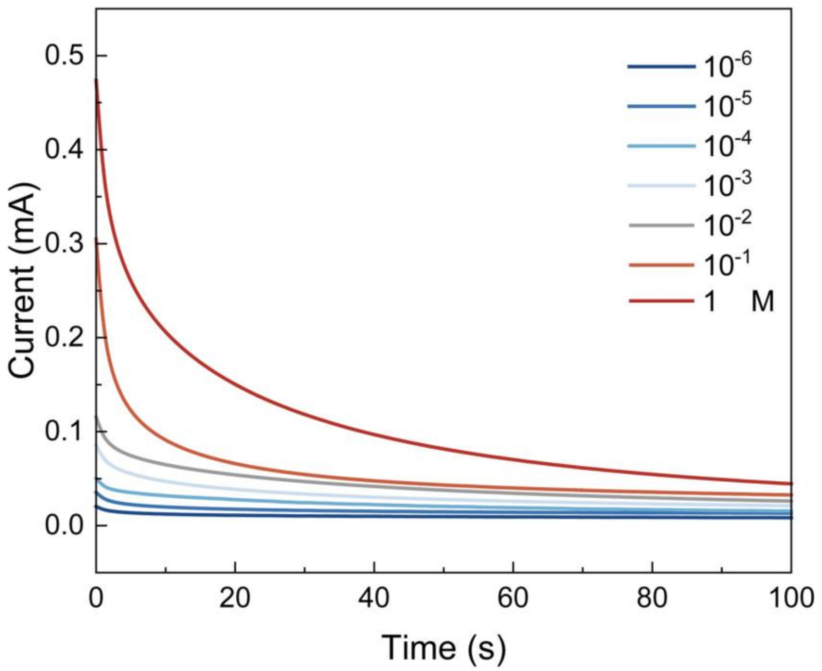

Supplementary Fig. 7. Time dependent current during the protonation process under different proton concentrations. A constant bias voltage of -0.7V is applied to the working electrode.

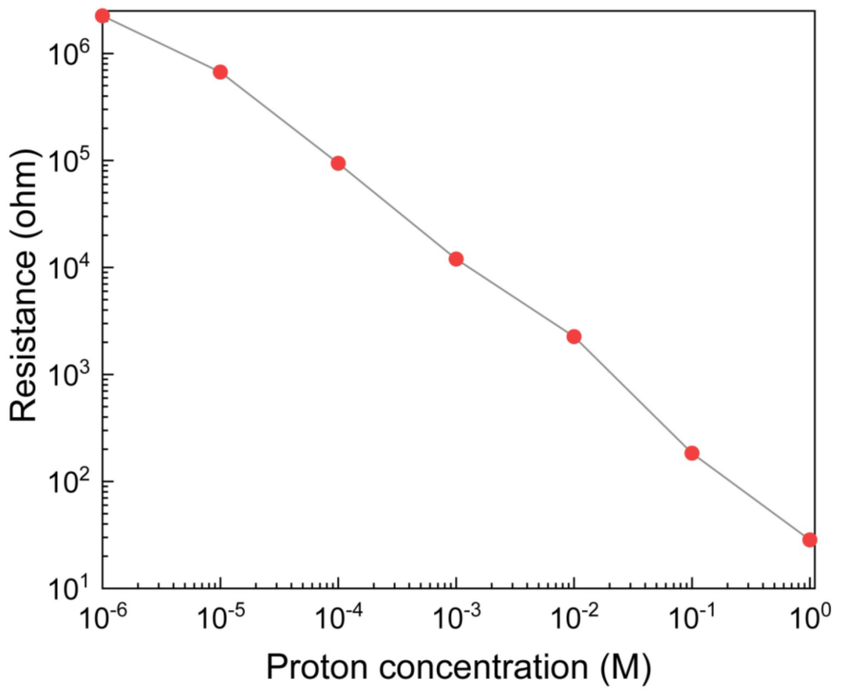

Supplementary Fig. 8. Proton concentration dependent proton solution resistance.

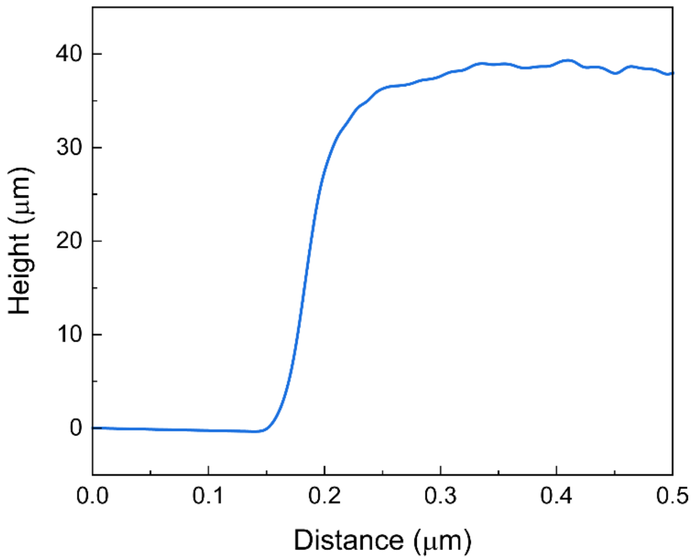

Supplementary Fig. 9. Profilometer measurement result for the electrode layer.

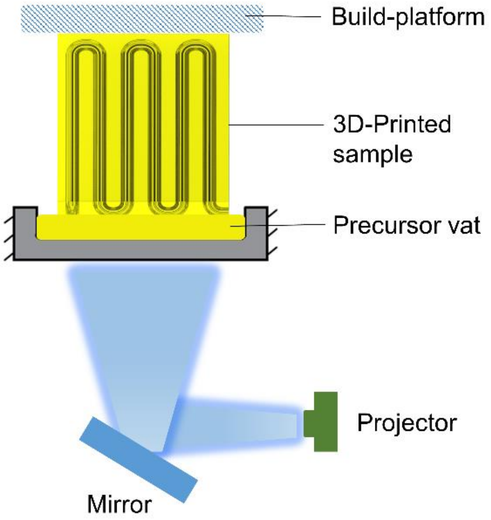

Supplementary Fig. 10. Schematic figure for 3D printing process.

The ultraviolet (UV) light was generated by the projector, and the control of image projection was achieved through a dynamic micro-mirror device (DMD, Texas Instruments). The automation and synchronization of the mask-image generation and the motion of the build platform were achieved using a custom-programmed control software[2]. A custom-made vat was fabricated using transparent acrylic sheets. The vat bottom was coated with polydimethylsiloxane (PDMS, DOW SYLGARD 184) to reduce the separation force on the cured sample[3].

Supplementary Table 1. EIS fitting reports.

Parameters	Discharging voltages		
	-0.5V	-0.55V	-0.6V
R_s (Ω)	6.4589	6.4507	6.4591
R_{ct} (Ω)	32.913	19.644	13.643
Capacitance (uF)	0.0001366	0.0000917	0.0000863

Supplementary References

1 Oates, C. *et al.* High field ferromagnetic resonance measurements of the anisotropy field of longitudinal recording thin-film media. *J. Appl. Phys.* **91**, 1417-1422 (2002).

2 Zhou, C., Chen, Y. & Waltz, R. A. Optimized mask image projection for solid freeform fabrication. *ASME J. Manuf. Sci. Eng.* **131** (2009).

3 Zhou, C., Chen, Y., Yang, Z. & Khoshnevis, B. Digital material fabrication using mask‐image‐projection‐based stereolithography. *Rapid Prototyp. J.* (2013).
