# Supplementary material for: Molecular magneto-ionic proton sensor in solid-state proton battery
Source: Nat Commun. 2022 Nov 17;13:7056. doi: 10.1038/s41467-022-34874-6 (PMC9672057; doi:10.1038/s41467-022-34874-6)
Supplement: Supplementary file 2 — Description of Additional Supplementary Files [file 41467_2022_34874_MOESM2_ESM.pdf]

Description of Additional Supplementary Files

File Name: Supplementary Movie 1
Description: The overall process of printing hydrogel with channels for solution to flow in and out.

File Name: Supplementary Movie 2
Description: Magnetic field distribution obtained through HFSS simulation.
